# The Nursing Supplement

**Published:** 1888-08-25

**Authors:** 


The Nursing Mirror.

All Contributions for this Supplement should be addressed "The Nursing Mirror," care of The Editor, 38, Old Jewry, E.C.

### En Passant.

A NATURAL SEQUENCE !—"The cholera was very bad in the town, and he had much to do with the work of providing doctors and nurses and coffins for the victims." —"Life of the Right Hon. W. E. Forster," vol. i. page 251.

POOR STUDENT !—*Nurse A.:* I do believe Mr. Smith has carried off the chloroform again. What on earth can he want with it? *Nurse B.:* I should think he wants to get a quiet night; and, if so, he must chloroform his clothes, or they would keep him awake. They are so dreadfully loud.

THE DUTY OF THANKFULNESS.—Nurses in hospitals are rather apt to lay too much stress on the advantages received by the patients and their duty of thankfulness, but still it is the poor soldier who suffers most from always having his causes to be grateful flung in his teeth. Witness the following true story: *Chaplain:* So poor Hopkins is dead. I should have liked to speak to him once again, and soothe his last moments; why didn't you call me? *Hospital Orderly:* I didn't think you ought to be disturbed for 'Opkins, sir, so I just soothed 'im as best I could myself. *Chaplain:* Why, what did you say to him? *Orderly:* "'Opkins," sez I, "you're mortal bad." "I am," sez 'e. "'Opkins," sez I, "I don't think you'll get better." "No," sez 'e. "'Opkins," sez I, "your're going fast." "Yes," sez 'e. "'Opkins," sez I, "I don't think you can 'ope to go to 'eaven." "I don't think I can, sez 'e. "Well then, 'Opkins," sez I, "you'll go to 'ell." "I suppose so," sez 'e. "Opkins," sez I, "you ought to be wery grateful as there's a place perwided for you, and that you've got somewhere to go." And I think 'e 'eard, sir, and then 'e died.

THE QUESTION OF UNIFORM.—In the life of W. E. Forster that has lately been published is a letter dated from Bucharest, in September, 1876. Mr. Forster tells how he has been to see Miss Johnston's five nurses who are having a special hospital, an old school, fitted up for them. They were then living in a quiet hotel and learning Servian. The war between the Turks and Servians was then waging, and Forster thought it was a very doubtful venture for these undoubted ladies to trust themselves amongst such a lot of cut-throats and adventurers as had joined both sides. He concludes: "Their dress seems to me most foolish, that of a maid-servant; not a dress that demands respect; not to be compared with the religious habit of the Catholic sisters." Now the question of uniform is one which is of certain consequence in the nursing world, and this denunciation of the usual simple and convenient dress seems remarkable, coming from such a source. The unwieldy garb of a Sister of Mercy, when viewed dispassionately, is both ugly and inconvenient, its only advantage being derived from association. Even the veil which an ordinary nurse attaches to her bonnet is often in the way and gets blown across the eyes, and the sweeping dress and long cap tails, which some hospital sisters wear, have their disadvantages. The question is whether a certain amount of comfort should be sacrificed for the sake of a more awe-inspiring uniform, or whether it is only necessary to be neatly dressed? It is not in any way a mere question of appearance for an ordinary woman to reply to, for she can know little of the wondrous power which a uniform seems to confer. Sights and duties from which we would shrink in ordinary garb we can face boldly when clad in uniform; and obedience and respect yielded to the nurse at once, are often denied to the lady who bears no outward badge of her profession. Mr. Forster's condemnation of a simple dress merits consideration.

TWO NOTES FROM AMERICA.—Miss Hampton, of South Carolina, a daughter of General Wade Hampton, is now assistant in the surgical ward of a New York hospital. She has taken a thorough course of training as a professional nurse, and it is her plan, when her studies are completed, to open a training school for nurses in the South, and supply a new field of work for Southern women. Dr. Kate I. Kelsey, of Menomonie, Wis., has been elected City Physician and Poor Commissioner for the third time. Her present re-election is unanimous.

WATCHING LUNATICS.—How often does a nurse who has charge of an insane person get into trouble by the escape of her patient! The cunning of lunatics is proverbial, and the trials of attending on them is known only to their nurses. One of the latest of such cases has come before a magistrate, for the patient is a ward in Chancery, and is believed to be hidden in the house of a relative. She was sent out walking with her nurse, and managed to slip away, in the unaccountable manner in which such things always occur. It must be an uncomfortable position for the nurse, to suddenly find herself minus a patient.

A CATECHISM FOR NURSES.—The August number of *Nursing Notes* contains an excellent supplement, consisting of a series of questions which every probationer should be able to answer at the end of her term of training. It forms a useful guide to nurses in showing them how much it is necessary to know, and if there are any questions they cannot answer, they can set to work at once to learn to do so. Have you done dry cupping? Have you administered the galvanic battery? Have you put on an extension? Have you seen a straight-jacket put on? Have you injected ergot? and many more questions might too often have to be answered in the negative by a certificated nurse. Chance may keep a nurse from learning some very simple necessity of her profession, and she may never notice her ignorance till she finds herself at a loss while attending a private case. Whereas, had she possessed this series of questions, she would have thought—No! I have never seen that done, I must ask matron to let me know when it next occurs.

COURAGE.—Lord Wolseley has written an article on "Courage" which is being much talked about, and the press has been busy in trying to top his examples of that virtue. With all due regard for the noble author and his critics, we still think that all the examples cited are of the lowest form of courage; mere brutal disregard of death, mere daring under the influence of momentary excitement and enthusiasm. This may be the prevailing form of courage amongst soldiers, but there is another form more often seen in women, especially in nurses. We refer to the cool courage of endurance, to the power of facing not sudden death, but lingering disease. It is the courage to bear contrasted with the courage to dare, and, in spite of the poet, we do not think these things are the same. During the Crimean war the surgeons got very little thanks for all they did, though one man was left alone and utterly unprotected amongst a crowd of wounded of both nationalities. The courage of the surgeon who performs a great operation, the courage of a nurse who works through a small-pox epidemic, are not these also worthy of a word of praise? No hope of honour or reward lured them on; no medals decorated their breasts; no public enthusiasm is for them. The long, trying labours of moral courage are forgotten in the sparkle of some dashing deed of mere physical prowess. We quote the last of Lord Wolseley's anecdotes as a specimen of the tone of the article: "I shall conclude this article with the story of the English general who, before he attacked Cadiz, thus addressed his men, 'You, Englishmen, who are fed upon beef, don't surely mean to be beaten by a —— lot of Spaniards, who live upon oranges!"

### Original Articles.

### A NURSE'S PROGRESS.—FROM APPLICATION TO CERTIFICATION.

### Paper III.—Certification.

The "school" side of nursing life is one which is very strange to the public, and many a probationer is astonished to find that she has classes and lectures to attend, and papers to write, and finally an examination to pass. Instead of a nurse leaning over a bed and holding the cup of cooling drink to the fevered lips, we have a vision before us of a frantic probationer, with her fingers in her ears, bent double over a dirty old copy of Kirke's "Handbook of Physiology;" and the second picture is as true as the first. It is the way of the world just now to demand of every class of human beings that they shall go in for this or that examination, and procure as many certificates as possible. Life, with too many of us, is a mad rush to pass examinations, a desire to be certified to possess much knowledge, an insane longing for stamped signed parchments, which can comfort no man's soul. And the matron of a hospital, when she sees the examination list, cannot help a sigh for that good little Nurse So-and-So, who is at the very bottom of the list, while that other loud-voiced, slap-dash nurse is at the very top. However, examinations are necessary, if not absolutely reliable, and the nurse must prepare for them by giving her best attention to lectures and classes. The lecture arrangements differ in the various hospitals. At King's College the medical superintendent lectures once a week, and the home-sister three times a week; but these last would rather be called classes in some institutions. At the London the matron gives a course of twelve lectures on general details of nursing, one of the surgeons gives a course on anatomy and surgical nursing, and one of the physicians a course on physiology and medical nursing. Besides these thirty-six lectures given each year there are two classes held every week by competent sisters, who overlook the notes taken at the lectures, and give instruction in bandaging, or any points the probationers wish explained. The nurse who wishes to succeed at the yearly examination must attend carefully to every lecture, and take notes of the same. It is trying, after a day's practical work, to sit down and follow an hour's theoretical instruction, but the effort has to be made.

A great aid to the thorough assimilation of these lectures and classes is to found in the reading of suitable books. Probationers are often too eager to attack learned or large works, instead of steadily studying the subject of the week's lecture. When the surgical course is proceeding, some book like Bell's "Surgery for Nurses," or Pye's "Elementary Bandaging and Surgical Dressing," and a primer of anatomy should be read; then, while the physician is lecturing, such books as Anderson's "Medical Nursing" and Huxley's "Physiology" can be taken up. Kirke and Quain are rather ponderous for a probationer in her first year, and more suitable for medical students. Slow and steady is the best motto for a nurse, for if her mind is too much given to scientific volumes, she may miss opportunities of learning in the ward which may never occur again. A note-book carried ever in the pocket, wherein all difficulties can be entered, is useful in this way; it teaches a nurse to observe and wish to understsnd, and at the weekly classes she can appeal to the sister to solve the problems which have perplexed her. In some training schools it is compulsory for every probationer to keep a diary for the home-sister's inspection, but where this is not the case a note-book answers very well. When possible a nurse should always have some acquaintance with a microscope, and its use in medicine. A few simple slides of various tissues are a great aid in comprehending lectures on physiology.

After a year, then, spent in practical experience in the wards, in attendance and lectures and classes, and in reading and observing as much as possible, the period for the dreaded examination at last approaches. Some twenty nurses are seated at intervals at the long tables, and before each is a sheet of blank paper and a list of twelve questions. An hour is given in which to answer at least nine questions correctly, and to take a prize it is necessary to answer them all. We constantly give in our pages specimens of examination questions set to probationers at the different hospitals, and perhaps the best private preparation for an examination is to try and answer these when time permits. The questions of course include nursing, anatomy, and physiology, and never turn on any subject which has not been discussed at the lectures. Taken quietly, they can generally be answered in the time allowed, but it is better to correctly reply to half than to give muddled solutions of the whole. The worst part of the examination is yet to come; both surgeon and physician like to put a few *viva-voce* questions, and see a temperature charted or a bandage done. To a nervous nurse this is very trying; the bandage won't reverse, the Latin words she is desired to translate won't tell their English equivalent to her. But the examiners are used to nervousness, and make all allowances; when the momentary fright is over the nurse does her work well, and retires at last; the dreaded examination is over.

The certificate is gained—at least we will hope so—and now what is the next step in a nurse's career, and what is this same certificate worth? If it is a good one and held by a woman who, besides being clever at an examination, is kind by a sick-bed, it often means a rise to the post of sister and thence to matron—heights to which we are unable to follow our successful probationer. But the first and simple result is that the holder of the certificate is now entitled to call herself a trained nurse; she is supposed to be competent to take charge of a ward, to go into a house as a private nurse, or to work for a district nursing association. At many hospitals, where the work is hard but the certificate highly valued, most of the nurses who pass their examination desert their alma mater immediately, and take up the more lucrative work of private nursing. Now where a certificate only takes two years to win, this is fair neither to the hospital nor to the nurse. The hospital has trained the nurse and taught her all she knows, and actually paid her to learn. A sense of loyalty should therefore inspire the nurse to remain a year as charge nurse if it is possible, and if the matron shows the slightest wish to detain her. Besides, in private work the sole control of all sorts of cases is in the nurse's hands; if there is any point beyond her there is no sister or matron to refer to, and the nurse will often wish she had not rashly undertaken such serious responsibilities. That extra year as head of a ward teaches a great deal to a woman, and if she is wise she will never leave the place of her training till three years have passed, if the authorities will keep her. The charge nurse is one step nearer to the sister, and the sister often becomes superintendent, and finally a matron.

What though private nursing may offer better pay for the moment and a change of scene, it has not the future to it that hospital work has. It is a very good experience for a nurse for a short time, but after a couple of years it is often found more irksome than hospital work, and less stimulating to the intellect. Now we have so many institutions for private nurses connected with our large hospitals, it ought to be possible to get a pleasant variety of ward and private work. Then the certificated nurse may enter on district nursing, may emigrate to India or Canada, or try a winter in the South of France amongst consumptive invalids. She has but to produce her certificate and be able to refer to the matron for a good moral character, and a hundred paths are thrown open to her, along which it is impossible to trace her progress. Only this is sure: if she have the elements of progress in her she will not long remain merely "nurse," she will secure some higher title, and pass on into realms whither these papers do not pretend to follow.

### Everybody's Opinion.

[*Correspondence on all subjects is invited. No communications can be entertained if the name and address of the correspondent is not given, or unless one side of the paper only be written on.*]

### CENSORIOUSNESS.

There is a fault, unhappily common even amongst professing Christians, which not only offends against the love that ought to be borne by one towards her sister, but actually tends to break the links which connect civil society. I allude to the unseemly practice of censoriously blaming the character and conduct of others. Peace and goodwill are frequently thus destroyed, friends are separated, and a scandal is raised even against religion itself. A very remarkable example of Christian forbearance once came under my personal knowledge. A beloved individual could never be moved to anything resembling indignation, except by an attempt to censure others. It could only be an attempt in her presence; for a censorious remark was sure to meet from her a prompt and effectual check. Let this be taken as a pattern for Christians, and those professing in general to follow. She that endeavours to bind up a wound is far better aware of the danger and extent of it than she that at a distance mocks at the sufferings of the wounded woman; and thus our blessed Lord exhibited in His own conduct in the closest union, a holy hatred of sin, which could be expiated only by His death, and a kind pity for the transgressors, in whose behalf he even prayed while they were murdering him, that that offence might be forgiven. We must follow His example in this respect. While from a knowledge of what sin has done to us we seek to view it with a perfect hatred, let us, with forbearing love, not presume to judge our sister; to her own Master she must stand or fall. Let us not be ready to find fault; let us mark the extenuating circumstances in her case; let us seek to rule our tongue, which is so ready to speak evil; and let every professing Christian strive not merely to be free from censoriousness herself, but to give no countenance to it in others. Nurse D. White.

### SMALL HOSPITALS FOR INEXPERIENCED PROBATIONERS.

Will you kindly give me a list of the cottage hospitals or small convalescent homes where they will take a probationer with no previous training? A friend of mine is very anxious to learn nursing, but is not strong enough for a London or even a local hospital; and it is so difficult to get hold of the names of small hospitals. Will you kindly help me?

L. M. Wills.

[Miss Wills will find a complete list of the institutions she inquires about in "Help in Sickness," by Henry C. Burdett (Kegan Paul, Trench, and Co., price 1*s.* 6*d.*), pages 33 to 46 and 121 to 126.—Ed. *T.H.*]

### THE CAUSE OF WEAK ANKLES.

"A Practical Mother" writes: "May I ask you to insert a few lines on a subject of great and growing importance, viz., the weak and deformed ankles of children? Taking a rest by the sea I have leisure and opportunity to observe how very many young girls and boys are affected in this way, or rather, how very few have strong, well-formed ankles. Several well-cared for ones wear iron supports, evidently to correct this defect. The prominent inner bones project abnormally, so that in walking they rub together, cutting through boots, stockings, and even skin. The beautiful arch of the foot, on which so much beauty and grace in walking depends, seems to be displaced and flattened, causing great irritation and pain, besides making impossible what would otherwise be only an ordinary good walk. I have had this subject forced upon me for the past ten years, and when my last little one was only a few days old it struck me that I had discovered the secret, when I saw the nurse half suspend the infant by the feet in changing it, which I believe is the usual attitude. When one thinks of the innumerable times this is done, and the then soft state of the bones and muscles, it seems to my physiologically uninformed mind that the mischief may be so accounted for. Also, would not the lifting being done by the nurse's left hand account for its being the child's right foot that is usually the worst, if not the only one injured?"

Aldeburgh-by-Sea.

### THE MERCHANT TAYLORS' CONVALESCENT HOME.

I am a constant reader of your valuable paper, The Hospital, and I frequently see in it inquiries for convalescent homes. I think it just possible that you may not know of the Merchant Taylors' Convalescent Home for Ladies at Bognor. I have lately been a patient there, and can speak in the highest terms of it. All the arrangements are excellent, and I have derived great benefit from my visit there. There is also a convalescent home for men, quite separate from the ladies' one, but belonging to the same company. Applicants for admission must apply to Mr. F. G. Faithfull, Merchant Taylors' Hall, Threadneedle Street, E.C.

A Private Nurse.

### A Touching Story of Gratitude.

A gentleman was walking, late one night, along a street in London, in which stands the hospital for sick children. There were three acrobats passing along, plodding wearily home to their miserable lodgings after their day's work: two of them were men, and they were carrying the ladders and poles with which they gave their performance in the streets whenever they could collect a crowd to look on. The third was a little boy in a clown's dress. He trotted wearily behind, very tired, and looking pale and sick. Just as they were passing the hospital the little lad's sad face brightened for a moment. He ran up the steps and dropped into the box attached to the door a little bit of paper.

It was found there next morning. It contained a sixpence, and on the paper was written, "For a sick child." The one who saw it afterwards ascertained, as he tells us, that the poor little waif, almost destitute, had been sick, and in his weary pilgrimage was, a year before, brought to this hospital, which had been a "House Beautiful" to him, for he was here cured of his bodily disease. Hands of kindness had ministered to him, words of kindness had been spoken to him, and he had left it, cured in body and whole in heart.

Someone in a crowd had to-day slipped a sixpence into his hand, and as he passed the hospital, his grateful little heart gave up for other child-sufferers "all the living that he had." It was all done so quietly, so noiselessly; but oh! believe me, the sound of that little coin falling into God's treasury that night, rose above the roar and din of this mighty city, and was heard with joy in the very presence of God Himself. —*Echoes.*

A Missing Hospital Nurse.—A lady applied last week for publicity respecting the disappearance of her daughter, who had been missing since August 6th. She had been a nurse at the Royal Hospital for Diseases of the Chest, City Road, which institution she left on the day mentioned, and she had not since been seen or heard of. Her initials were E. M. W., her age was 28, she was 5 ft. 5 in. in height, was near-sighted, and of late had been desponding. She was dressed in black dress, waterproof cloak, black bonnet, long veil.

### Notes and Queries.

To Correspondents.—1. Questions may be written on post-cards. 2. Advertisements in disguise are inadmissible. 3. In answering a query please quote the number. 4. If a private answer is desired, a stamped addressed envelope must be forwarded.

(46) *Poor Law Infirmaries and Medical Students.*—1. E. G. wishes to know whether Medical Students "Walk" the Poor Law Infirmaries. 2. Also, if each infirmary has a lying-in ward?

**Answers.**

*Free Training in Midwifery.*—In answer to A.M.'s request for above, may I quote the following from your issue of July 28th, 1888.: "Female Hospital, Woolwich. Wanted, at once, a nurse. *Free training in Midwifery given.* Apply to the Matron." Cannot A. M. apply here.

(44) Nurse A can buy turn-down collars (The Squire) at any drapers. There is a special house which supplies bonnets, cloaks, &c., as worn by Bart.'s nurses, but I have forgotten address. I rather think it is Tarn's; but Nurse Marsh, 77, Thornlaw Road, West Norwood, S.E., could supply correct information. Nurse Waller.

(46) *Poor Law Infirmaries and Medical Students.*—Medical Students are not as a rule admitted to Poor-Law Infirmaries for clinical instruction. Lying-in Wards are attached to the workhouses in the majority of cases.

### Examination Questions.

[Copies of similar questions will be gladly received by the Editor.]

The following questions were given to probationers at the largest metropolitan hospital:—

i. How would you make and change the bed for a helpless patient? How would you prepare the bed for a case of Ovariotomy? Name any particular arrangement of the bed or bedding that you would consider desirable in any special cases you can think of.

ii. State the main points to be attended to in the Dietary of—(*a*) An infant brought up by hand. (*b*) A case of Ulcer of the Stomach, and give reasons for your statements.

iii. What should a nurse especially observe when in charge of the following cases?—Obstruction of the Bowels. Amputation of a Limb. Operation for Cataract. Severe burn.

iv. What points are important in the preparation and administration of the various kinds of Enemata? State any details you think a nurse should bear in mind in connexon with the various methods of administering drugs.

v. You are sent to a private house to nurse a patient suffering from Scarlet Fever: State shortly but definitely, with your reasons, what you consider of most importance—(*a*) When you undertake the case. (*b*) While you are nursing it. (*c*) When you leave it.

vi. State what you know of the structure of Bone; of the construction of a Joint; of the arrangement of Arteries and Veins; of the mode of Healing of Wounds; and of the Repair of a Broken Bone.

### Vacancies.

The following vacancies are announced:—

**Lady Superintendents.**

Leicester Institution of Trained Nurses; £40.
Institution of Trained Nurses, Leicester; £40.
West Kent General Hospital, Maidstone; £60.

**Matron.**

Lytham Cottage Hospital.

**Nurses.**

Bradford Infirmary; night; £25.

Bristol Royal Infirmary; £25 to £28.

Leeds, Borough Fever Hospital; two; £25.

Royal Albert Hospital, Devonport; two; £25.

Suffolk General Hospital, Bury St. Edmunds; £30.

Nursing Institute, Ryde.

Hull Sanatorium; four; £30.

Nottingham and Notts Nursing Institution; £25.

